# Supplementary material for: Association study of human leukocyte antigen (HLA) variants and idiopathic pulmonary fibrosis
Source: medRxiv. 2023 Jul 24:2023.07.20.23292940. Preprint. [Version 1] doi: 10.1101/2023.07.20.23292940 (PMC10402235; doi:10.1101/2023.07.20.23292940)

Association study of human leukocyte antigen (HLA) variants and idiopathic pulmonary fibrosis

Supplemental material

Beatriz Guillen-Guio*, Megan L. Paynton*, Richard J. Allen, Daniel P.W. Chin, Lauren J. Donoghue, Amy Stockwell, Olivia C. Leavy, Tamara Hernandez-Beeftink, Carl Reynolds, Paul Cullinan, Fernando Martinez, CleanUP-IPF Investigators of the Pulmonary Trials Cooperative, Helen L. Booth, William A. Fahy, Ian P. Hall, Simon P. Hart, Mike R. Hill, Nik Hirani, Richard B. Hubbard, Robin J. McAnulty, Ann B. Millar, Vidya Navaratnam, Eunice Oballa, Helen Parfrey, Gauri Saini, Ian Sayers, Martin D. Tobin, Moira K. B. Whyte, Ayodeji Adegunsoye, Naftali Kaminski, Shwu-Fan Ma, Mary E. Strek, Yingze Zhang, Tasha E. Fingerlin, Maria Molina-Molina, Margaret Neighbors, X. Rebecca Sheng, Justin M. Oldham, Toby M. Maher, Philip L. Molyneaux, Carlos Flores, Imre Noth, David A. Schwartz, Brian L. Yaspan, R. Gisli Jenkins, Louise V. Wain[#], Edward J. Hollox[#].

*Equal contribution as first authors
[#]Equal contribution as senior authors

METHODS

Study Cohorts

We analysed genomic data from seven previously described independent case-control studies of idiopathic pulmonary fibrosis (IPF). In all seven studies, IPF cases were diagnosed according to the American Thoracic Society and European Respiratory Society guidelines (1,2).

The Study of Clinical Efficacy of Antimicrobial Therapy Strategy Using Pragmatic Design in Idiopathic Pulmonary Fibrosis - University of California Davis (CleanUP-UCD) comprised a total of 469 IPF cases from a randomized clinical trial conducted across 35 US locations and from the University of California Davis (3,4), as well as 2,455 population controls selected from UK Biobank. Both cases and controls were genotyped using the Affymetrix UK Biobank array.

The Colorado study (5) included 1,515 patients with fibrotic idiopathic interstitial pneumonias (fIIP) from US cohorts (National Jewish Health IIP population, InterMune IPF trials, UCSF, Vanderbilt University IIP population, and the National Heart Lung and Blood Institute Lung Tissue Research Consortium) and 2,455 population controls selected so they were genetically similar to the cases. All individuals were genotyped using the Illumina Human 660W Quad BeadChip array.

The Genentech study (6,7) consisted of 813 cases from three IPF clinical trials (ASCEND, CAPACITY, and RIFF) and 3,949 controls from non-IPF clinical trials for age-related macular degeneration, diabetic macular oedema, multiple sclerosis, asthma, and inflammatory bowel disease. Genotypes were obtained from whole-genome sequencing using HiSeq X Ten platform (Illumina Inc.) to an average read depth of 30X.

The Idiopathic Pulmonary Fibrosis Job Exposures Study (IPF-JES) (8) included 416 men from England, Scotland or Wales diagnosed with IPF and 2,465 matching controls (all men) selected from both the IPF-JES study (individuals with an outpatient clinic appointment during the study period) and UK Biobank. Genotyping was performed with the Affymetrix UK Biobank array.

The UK study (9) comprised a total of 612 IPF cases from 9 different centres across the UK and 3,366 matching controls selected from UK Biobank. Cases were genotyped with the Affymetrix UK BILEVE array and controls with the UK Biobank array.

The US study (10) included 541 IPF cases from the University of Chicago, University of Pittsburgh and COMET study, and 542 population controls from the database of genotypes and phenotypes (dbGaP) and the University of Pittsburgh, all genotyped with the Affymetrix Genome-Wide Human SNP 6.0 array.

The UUS study [United States, United Kingdom, and Spain] (11) consisted of 793 IPF cases from 7 study cohorts (ACE, PANTHER, UCD, Chicago, UCSF, PROFILE, and Spain) and 9,999 population controls selected from UK Biobank matching ancestry, sex and smoking distribution. Cases were genotyped with the Affymetrix UK Biobank and Spain Biobank arrays, and controls with the UK Biobank array.

All studies were performed in accordance with The Code of Ethics of the World Medical Association (Declaration of Helsinki) and approved by the appropriate institutional review or Research Ethics Committee.

REFERENCES

1. Raghu G, Remy-Jardin M, Myers JL, et al. Diagnosis of Idiopathic Pulmonary Fibrosis. An Official ATS/ERS/JRS/ALAT Clinical Practice Guideline. *Am J Respir Crit Care Med* 2018;**198**(5):e44-e68.
2. Raghu G, Collard HR, Egan JJ, et al. An official ATS/ERS/JRS/ALAT statement: idiopathic pulmonary fibrosis: evidence-based guidelines for diagnosis and management. *Am J Respir Crit Care Med* 2011;**183**(6):788-824.
3. Martinez FJ, Yow E, Flaherty KR, et al. Effect of Antimicrobial Therapy on Respiratory Hospitalization or Death in Adults With Idiopathic Pulmonary Fibrosis: The CleanUP-IPF Randomized Clinical Trial. *JAMA* 2021;**325**(18):1841-51.
4. Allen RJ, Oldham JM, Jenkins DA, et al. Longitudinal lung function and gas transfer in individuals with idiopathic pulmonary fibrosis: a genome-wide association study. *Lancet Respir Med* 2023;**11**(1):65-73.
5. Fingerlin TE, Murphy E, Zhang W, et al. Genome-wide association study identifies multiple susceptibility loci for pulmonary fibrosis. *Nat Genet* 2013;**45**(6):613-20.
6. Dressen A, Abbas AR, Cabanski C, et al. Analysis of protein-altering variants in telomerase genes and their association with MUC5B common variant status in patients with idiopathic pulmonary fibrosis: a candidate gene sequencing study. *Lancet Respir Med* 2018;**6**(8):603-14.
7. Donoghue LJ, Stockwell AD, Neighbors M, et al. Identification of a Genetic Susceptibility Locus for Idiopathic Pulmonary Fibrosis in the 16p Subtelomere Using Whole-Genome Sequencing. *Am J Respir Crit Care Med* 2023;**207**(7):941-44.
8. Reynolds CJ, Sisodia R, Barber C, et al. What role for asbestos in idiopathic pulmonary fibrosis? Findings from the IPF job exposures case-control study. *Occup Environ Med* 2023;**80**(2):97-103.
9. Allen RJ, Porte J, Braybrooke R, et al. Genetic variants associated with susceptibility to idiopathic pulmonary fibrosis in people of European ancestry: a genome-wide association study. *Lancet Respir Med* 2017;**5**(11):869-80.
10. Noth I, Zhang Y, Ma SF, et al. Genetic variants associated with idiopathic pulmonary fibrosis susceptibility and mortality: a genome-wide association study. *Lancet Respir Med* 2013;**1**(4):309-17.
11. Allen RJ, Guillen-Guio B, Oldham JM, et al. Genome-Wide Association Study of Susceptibility to Idiopathic Pulmonary Fibrosis. *Am J Respir Crit Care Med* 2020;**201**(5):564-74.

SUPPLEMENTARY TABLES

Table S1: Sample size of the study

	Total	Cases	Controls
CleanUP-UCD	2,924	469	2,455
Colorado	6,198	1,515	4,683
Genentech	4,762	813	3,949
IPF-JES	2,881	416	2,465
UK	3,978	612	3,366
US	1,083	541	542
UUS	10,792	793	9,999
**Total**	**32,618**	**5,159**	**27,459**

The number of individuals shown is the result after quality controls.

Table S2: Effect allele frequencies in each study

Variant ID	Position (b38)	NEA/EA	EAfreq Meta	EAfreq / Imp.Q						
				CleanUP-UCD	Colorado	Genentech	IPF-JES	UK	US	UUS
rs4947344	chr6:32710069	C/T	0.286	0.293 / 0.991	0.290 / 0.987	0.281 / NA	0.281 / 0.991	0.283 / 0.997	0.290 / 0.984	0.280 / 0.992
rs9266618	chr6:31378389	A/C	0.093	0.100 / 0.979	0.083 / 0.959	0.083 / NA	0.097 / 0.979	0.103 / 0.983	0.084 / 0.898	0.102 / 0.972
rs116450899	chr6:28764432	G/A	0.089	0.092 / 0.999	0.093 / 0.998	0.083 / NA	0.088 / 0.999	0.091 / 0.999	0.090 / 0.996	0.087 / 0.999
rs138188178	chr6:30632465	G/T	0.043	0.044 / 0.989	0.044 / 0.99	0.046 / NA	0.04 / 0.985	0.043 / 0.995	0.036 / 0.894	0.043 / 0.987

NEA, non-effect allele; EA, effect allele; EAfreq, frequency of the effect allele. Imp.Q, Imputation quality.

Table S3: Sensitivity analyses of sentinel variants excluding the Colorado study

Variant ID	Position (b38)	NEA/EA	Results excluding Colorado		
			OR [95%CI]	P	PPR
rs4947344	chr6:32710069	C/T	1.13 [1.07,1.20]	$2.43x10^{-5}$	1.24%
rs9266618	chr6:31378389	A/C	1.17 [1.07,1.28]	$3.52x10^{-4}$	0.63%
rs116450899	chr6:28764432	G/A	1.18 [1.08,1.29]	$2.83x10^{-4}$	1.88%
rs138188178	chr6:30632465	G/T	1.23 [1.08,1.39]	$1.10x10^{-3}$	0.88%

NEA, non-effect allele; EA, effect allele. Associations reaching the Bonferroni threshold (*p*=$4.50x10^{-4}$) are shaded in grey. PPR, posterior probability of replication (obtained with MAMBA, Meta-Analysis Model-Based Assessment of Replicability).

Table S4: Validation of the *HLA-DQB1*06:02* association with IPF

Variant ID	Position (b38)	Meta-analysis			Excluding Colorado		
		OR [95%CI]	P	PPR	OR [95%CI]	P	PPR
*HLA-DQB1**06:02	chr6:32663284	1.16 [1.09, 1.23]	$5.66X10^{-6}$	0.64%	1.08 [1.00, 1.16]	0.043	0.09%

PPR, posterior probability of replication (obtained with MAMBA, Meta-Analysis Model-Based Assessment of Replicability).

SUPPLEMENTARY FIGURES

Figure S1. Conditional analysis on rs4947344

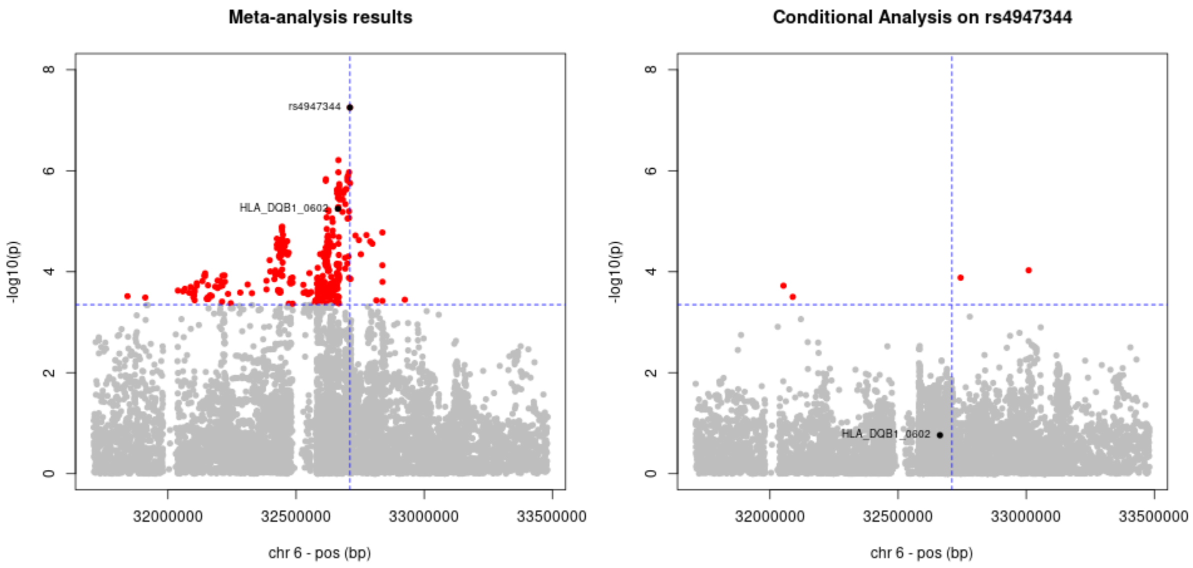

Figure S2. Forest plot of the validation analysis results for *HLA-DQB1*06:02*

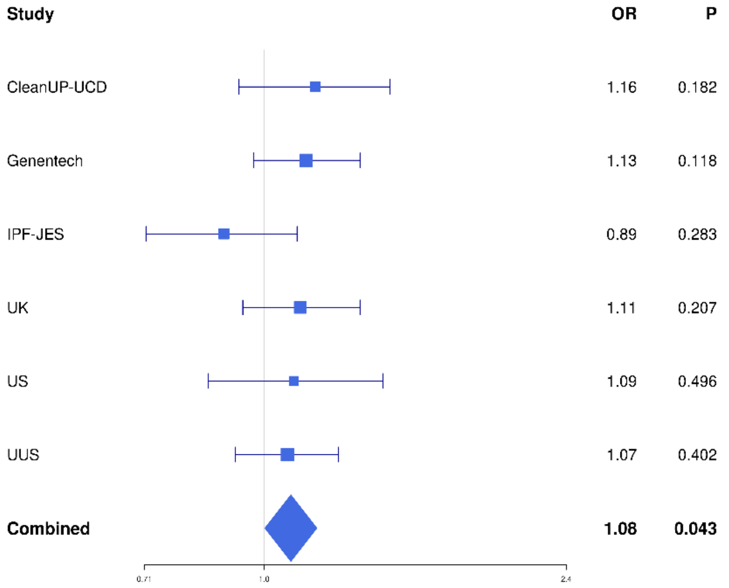

Supplement: Supplement 1 [file media-1.pdf]
